# Haplotypes in the *GC*, *CYP2R1* and *CYP24A1* Genes and Biomarkers of Bone Mineral Metabolism in Older Adults

**DOI:** 10.3390/nu14020259

**Published:** 2022-01-08

**Authors:** Ana Fernández-Araque, Andrea Giaquinta-Aranda, Carmelo Moreno-Sainz, María Cruz Martínez-Martínez, Verónica Velasco-González, María Sainz-Gil, Luis H. Martín-Arias, Silvia Carretero-Molinero, Miguel García-Hidalgo, Zoraida Verde

**Affiliations:** 1 Department of Nursery, Campus Duques de Soria, University of Valladolid, 42004 Soria, Spain; afa@enf.uva.es (A.F.-A.); agaranda1993@hotmail.com (A.G.-A.); silvia.carretero@uva.es (S.C.-M.); 2 Grupo de Investigación Reconocido “Pharmacogenetics, Cancer Genetics, Genetic, Polymorphisms and Pharmacoepidemiology”, University of Valladolid, 47005 Valladolid, Spain; veronica.velasco.gonzalez@uva.es (V.V.-G.); maria.sainz@uva.es (M.S.-G.); luish.martin@uva.es (L.H.M.-A.); 3 Department of Hemodialysis, Hospital Santa Bárbara, 42005 Soria, Spain; 4 Department of Clinic Biochemistry, Hospital Santa Bárbara, 42005 Soria, Spain; cmorenos@saludcastillayleon.es (C.M.-S.); mcmartinezma@saludcastillayleon.es (M.C.M.-M.); 5 Department of Nursery, University of Valladolid, 47005 Valladolid, Spain; 6 Centro de Farmacovigilancia de Castilla y León, University of Valladolid, 47005 Valladolid, Spain; 7 Centro de Estudios sobre la Seguridad de los Medicamentos (CESME), University of Valladolid, 47005 Valladolid, Spain; 8 EiFAB-iuFOR, Campus Duques de Soria, University of Valladolid, 42004 Soria, Spain; miguel.garcia.hidalgo@uva.es; 9 Departamento de Bioquímica, Biología Molecular y Fisiología, Campus Duques de Soria, University of Valladolid, 42004 Soria, Spain

**Keywords:** elderly, genetic polymorphisms, 25 hydroxy vitamin D, albumin, calcium, phosphorus, intact parathyroid hormone, *GC*, *CYP2R1*, *CYP24A1*

## Abstract

Candidate gene studies have analyzed the effect of specific vitamin D pathway genes on vitamin D availability; however, it is not clear whether genetic variants also affect overall bone metabolism. This study evaluated the association between genetic polymorphisms in *GC*, *CYP2R1* and *CYP24A1* and serum levels of total 25(OH)D, iPTH and other mineral metabolism biomarkers (albumin, total calcium and phosphorus) in a sample of 273 older Spanish adults. We observed a significant difference between *CYP2R1* rs10741657 codominant model and total 25(OH)D levels after adjusting them by gender (*p* = 0.024). In addition, the two SNPs in the *GC* gene (rs4588 and rs2282679) were identified significantly associated with iPTH and creatinine serum levels. In the case of phosphorus, we observed an association with *GC* SNPs in dominant model. We found a relationship between haplotype 2 and 25(OH)D levels, haplotype 4 and iPTH serum levels and haplotype 7 and phosphorus levels. In conclusion, genetic variants in *CYP2R1* and *GC* could be predictive of 25(OH)D and iPTH serum levels, respectively, in older Caucasian adults. The current study confirmed the role of iPTH as one of the most sensitive biomarkers of vitamin D activity in vivo.

## 1. Introduction

Vitamin D deficiency is a global health problem. Interindividual variability is attributable to several factors such as sun exposure, geographical latitude, dietary intake or inherited characters, being approximately 53% of this variability due to them. While vitamin D deficiency is an important public health topic, at least some vitamin D-related gene polymorphisms seem to play an important role in vitamin D status [1,2]. Apart from the functional vitamin D insufficiency caused by several rare mendelian disorders, there are several candidate genes in the specific vitamin D pathway [3,4,5,6,7].

The musculoskeletal consequences of lower vitamin D concentrations affecting to mineral and bone metabolism are well known, such as rickets, fractures and osteomalacia [8]. However, more extra-skeletal effects of vitamin D have been revealed in the last decade, (e.g., increased risk of chronic diseases as cardiovascular disease, diabetes or dementia) [9,10,11].

Vitamin D is a prohormone whose active metabolite, 1,25-dihydroxyvitamin D (1,25(OH)2D), in association with parathyroid hormone (PTH) and total calcium, regulating calcium homeostasis and playing an important role in bone growth and remodeling [12]. Vitamin D lower concentration would affect bone metabolism by decreasing total calcium absorption, with a secondary increment in PTH secretion, which may lead to bone resorption rising [13,14]. PTH also stimulates the conversion of 25-hydroxyvitamin D (25(OH)D) into the active form (1,25(OH)_2_D) and enhance calcium absorption in the intestine.

Vitamin D is mainly produced in the skin by exposure to sunlight. The two most important forms of vitamin D are vitamin D3 (cholecalciferol) and vitamin D2 (ergocalciferol). In contrast to vitamin D3, the human body cannot produce vitamin D2, which is taken up with fortified food or given by supplements. In humans, plasma vitamin D3 and D2 are bound to the vitamin D binding protein and transported to the liver where both are hydroxylated to form 2 [12,13,14,15]. It is biologically inert and must undergo two successive hydroxylations in the liver and kidney to become the biologically active 1,25(OH)_2_D.

Vitamin D metabolism is highly regulated; variation in expression or activity of key proteins may modify its level or effects. Vitamin D is metabolized first to 25(OH)D by the hepatic 25(OH)D-hydroxylases [16]. The 25(OH)D-hydroxylase enzyme, encoded by the cytochrome P450 family 2 subfamily R member 1 (*CYP2R1*) gene, has been shown as a key enzyme for the conversion of vitamin D into the form 25(OH)D in the liver and the cytochrome P450 family 27 subfamily B member 1 (CYP27B1) is the key 1-hydroxylase [17]. Finally, cytochrome P450 family 24 subfamily A member 1 (CYP24A1) catabolizes 25(OH)D as well as 1,25(OH)_2_D, into biologically inactive form [18]. Variants of *CYP2R1* and *CYP24A1* genes have been related to levels of vitamin D in several studies [3,19,20,21,22].

Serum 25(OH)D level is considered one of the most accurate indicators of vitamin D status (cutaneous synthesis and nutritional intake). It is the major form of circulating inactive vitamin D (with levels approximately 1000-fold greater than the circulating 1,25(OH)_2_D) and is commonly agreed that 25(OH)D is the metabolite to determine the overall vitamin D status as it is the major storage form of vitamin D in the human body. Most of the 25(OH)D, measurable in serum, is 25(OH)D3 whereas 25(OH)D2 reaches measurable levels only in patients taking vitamin D2 supplements and it is considered to be less effective [15].

The optimal circulating 25(OH)D level has been a matter of great debate over the last years as the Institute of Medicine Recommendations (IOM) [23]. Categorized people as “appropriate” is complicated by disparities in 25(OH)D levels in several populations (age, race or sex) [24,25].

Approximately 90% of 25(OH)D is bound to vitamin D–binding protein (DBP), and the remainder to albumin, less than 1% of 25(OH)D is free in plasma as lipophilic hormone [26]. Genetic variation in the DBP gene, *GC*, is associated with 25(OH)D levels [3,4]. Several genetic variants in *GC* gene are known to modulate DBP levels and have affinity for 25(OH)D [27]. However, there are very few studies about the effect of variants in *GC* in PTH level as one of the most sensitive biomarkers of vitamin D activity in vivo [28,29].

To date, most of published studies have focused on the association between vitamin D levels and genetic variants in specific unhealthy populations (osteoporosis, some cancers, autoimmune disease or risk of hypertension [15,30]. Moreover, it is not clear how other markers of bone metabolism as PTH in addition to 25(OH)D, as well as other biochemical markers, are influenced by genetic polymorphism in vitamin D pathway related genes [31,32].

Given the established risk of vitamin D deficiency for bone health and potential risks for major non-skeletal diseases, it is important to understand the role genetic factors play in modulating vitamin D levels. In previous candidate gene and genome-wide association studies, common single nucleotide polymorphisms (SNPs) in vitamin D pathway genes have been associated with circulating [25(OH)D]) [24,25,26,27].

The aim of the present study was to examine the association between polymorphisms and haplotypes of *GC*, *CYP2R1* or *CYP24A1* and levels of total 25(OH)D and PTH as well as other mineral metabolism biomarkers (albumin, creatinine, total calcium and phosphorus) in Spanish population over 65 years old.

## 2. Materials and Methods

### 2.1. Study Design

This study was a cross-sectional cohort study conducted from January 2018 to May 2018 and from January 2019 to May 2019. The study protocol was approved by the Local Ethics Committee of Area de Salud de Burgos y Soria (Ref. CEIC 1446) and conducted according to the guidelines laid down in the Declaration of Helsinki. A sample of Caucasian individuals aged 65 and older in the north of Spain (Soria) was screened for participation. Inclusion criteria were: aged more than 65 years old, not institutionalized, without diagnosis of renal illness, dementia, mobility impairments or chronic disorders that could affect bone mineral metabolism (as osteoporosis). Mini Mental Status Exam (MMSE) was used to screen for possible cognitive issues and mobility was evaluated assessing their ability to walk without any aid for more than one minute. Written informed consent was signed prior to testing.

### 2.2. Procedures

Two hundred and eighty-four elderly people (older than 65 years old) were recruited for the study. 

Selected participants who have visited several primary healthcare centers for routine medical check-up were interviewed by a research nurse collecting the following data: demographics, anthropometrics, smoking status, drugs prescribed, sun exposure, dietary supplement use and Clinical Group Risk (CGR) category. Sun exposure was considered ≥2 h day. Participants were considered vitamin D supplemented with a daily intake of 600 IU/day. The Barthel Index (BI), which is a recognized and simple scoring instrument, was used to evaluate basic activities of daily life (ADL) functions, the level of physical performance, and the intensity of needed care [33]. The BI is the most comprehensive tool to assess the physical condition and ADL impairment in elderly people. ADL assessment is a good proxy for a patient’s general health condition, comorbidity and risk of mortality [34].

#### 2.2.1. Biochemical Blood Analysis

Blood samples were obtained by venipuncture in the morning by a trained nurse. Blood samples were sent to the Hospital Santa Bárbara Biochemistry Service. Bone mineral metabolism biomarkers (serum total calcium, phosphorus, intact parathyroid hormone (iPTH), albumin, creatinine and 25-hydroxyvitamin D (25-OH-D) levels) were analyzed as previously published [35]. 

To classify the vitamin D levels (sufficient, deficient, insufficient), we followed the IOM cut-off points recommendations 2011 where vitamin D deficiency is defined as serum 25(OH)D levels below 12 ng/mL, vitamin D inadequacy is defined as 25(OH)D levels between 12 and 20 ng/mL and sufficiency is defined as serum 25(OH)D levels more than 20 ng/mL [10]. 

#### 2.2.2. Genotyping

Genomic DNA was isolated from EDTA blood tubes using a specific kit QIA Symphony DSP DNA Midi kit (Qiagen, Hilden, Germany) following the manufacturer’s recommendations. The extracted DNA was used to amplify sequences containing polymorphisms related to vitamin D. The following SNPs were selected because of the evidence of significant associations in previous large sample size studies in European-ancestry population. These SNPs included: *rs4588*, *rs2282679*, in *GC* gene, *rs10741657* in *CYP2R1* gene and *rs6013897* in *CYP24A1* gene. Selected SNPs were determined by real time polymerase chain reaction (RT-PCR) with TaqMan Probes (Thermo Fisher Scientific, Waltham, MA, USA).

### 2.3. Statistical Analyses

Characteristics of the participants were described as mean and standard deviation (SD) for continuous variables and frequencies (percentages) for categorical data. Student’s t-test or analysis of variance was used for continuous variables, and the Chi-square test was used for categorical variables. Associations between continuous variables of interest were tested with the Pearson Correlation. All statistical assessments were two-sided and considered to be significant when *p*-value was <0.05.

Data were analyzed using PASW/SPSS Statistics 24.0 (SPSS Inc, Chicago, IL, USA) program.

On the other hand, genetic data analysis was performed using the packages snpassoc and haplo.stats in R [36,37,38]. SNPs in Hardy–Weinberg Equilibrium according Wiggington and cols. were included in the association tests for different genetic models [39]. Association tests were computed for each genotype obtaining *p*-value with comparison with the null model and the Akaike Information Criterion (AIC).

Haplotypes were estimated by Expectation–Maximization algorithm and tested for association studies performing an iterative generalized lineal model regression to calculate regression coefficients and probabilities. The differences between groups at CI-95% were considered when *p* < 0.05, corrected in case of multiple comparison.

## 3. Results

In total, 284 subjects were screened for participation in the study, two of them were excluded due to creatinine levels greater than 1.9 mg/dL, total calcium > 10.9 mg/dL or phosphorus < 2.5 mg/dL. In addition, nine subjects were not included due to lost and missing data.

### 3.1. Study Subjects’ Characteristics

The mean age was 76.13 ± 7.09 years (range: 65–94 years) and 46.8% of patients recruited were men. Among the study subjects, 25.4% were in the normal range for body mass index (BMI), 49.5% were in the overweight range and 25.1% were obese. Approximately, 95% of the participants were non-smokers, 64% of the study population was exposed to the sun more than 2 h per day and 15% consumed dietary vitamin D supplement. In the case of BI, the mean was near 90 percent, which can be considered independent for ADL.

Attending the mineral metabolism biomarkers, the mean serum 25(OH)D level was 18.40 ± 8.89 ng/mL (range 3.00–68.46 ng/mL) and the mean serum iPTH concentration 66.08 ± 28.67 ng/L (range 19.84–185.80 ng/L). According to IOM cut-off points, 24.0% of the subjects enrolled were classified as vitamin D deficient, 40.6% insufficient and 35.4% adequate.

Women and men differed significantly in serum levels of total calcium, phosphorus or creatinine, (*p* = 0.005, *p* < 0.001 and *p* < 0.001, respectively) (see Table 1). 

Table 2 presents the results of Pearson’s correlation between crude variables. As it was expected, a strong correlation was present between age and BI mean, creatinine, albumin or iPTH (*p* < 0.001, *p* < 0.001 *p* < 0.001 and *p* < 0.001, respectively). In addition, we observed a marginal negative correlation between age and 25(OH)D or phosphorus (*p* = 0.062 and *p* = 0.099, respectively). Similar negative correlations were also observed between iPTH and total 25(OH)D or albumin *(p* < 0.001 and *p* < 0.001, respectively) and a positive correlation between iPTH and creatinine (*p* < 0.001).

The age and gender variables were considered potential confounding factors and were controlled in genetic association analysis.

### 3.2. Relationship between Genetic Variants and Mineral Metabolism Biomarkers

The allele frequencies of the 4 SNPs assessed in this study were in Hardy–Weinberg Equilibrium (HWE) and are reported in Table 3. 

We observed a significant difference between rs10741657 codominant model and total 25(OH)D levels after adjusting by gender (*p =* 0.024). In addition, the two SNPs in the *GC* gene (rs4588 and rs2282679) were significantly associated with iPTH and creatinine serum levels. In the case of phosphorus, we observed an association with *GC* SNPs in dominant model (see Table 4). Due to the LD association, we found the same association for both SNPs. The strongest association was observed for creatinine levels (*p* = 0.009 and *p* = 0.009; respectively), probably due to the combined effect of gender and iPTH influence in creatinine levels.

To analyze the combined effect of *GC-rs4588*, *GC-rs2282679*, *CYP2R1-rs10741657* and *CYP24A1-rs6013897* we generated several haplotypes (see Table 5).

Presence of the eight most common haplotypes was found in the 99.26% of the sample and we classified as rare haplotypes combinations with prevalence less than 1%. 

By haplotype analyses, attending to the most prevalent combinations, we found four haplotype block candidates (Table 6 and Table 7). Thus, we found relationships between haplotype 2 and 25(OH)D levels, haplotype 4 and iPTH serum levels, haplotype 5 and creatinine levels (marginal association) and haplotype 7 and phosphorus levels.

## 4. Discussion

In the present study, we investigated the association of four candidate SNPs with mineral metabolism markers levels in a group of 273 elderly subjects representative of the healthy age-related Spanish population. 

Consistently with prior studies and the known inhibitory effect of 25(OH)D on PTH production, our group showed an inverse correlation between baseline iPTH levels and total 25(OH)D. In addition, we observed a positive correlation between albumin levels and iPTH, the binding of 25(OH)D to albumin may modulate its physiological activity [28].

Vitamin D is mainly metabolized in the liver to 25(OH)D by CYP2R1 [16]. Later, 25(OH)D is transported by DBP, encoded by the *CG* gene, to the kidney. We have identified an association between *CYP2R1 rs10741657* and 25(OH)D concentration, which is consistent with previous studies [3,4,20,21,40]. Although several enzymes with 25-hydroxylase activity are involved in the 25-hydroxylation of vitamin D, CYP2R1 activity is critical at this first step in vitamin D metabolism [3,16,41]. In addition, *CYP2R1* variants cause vitamin D related pathologies as rickets as human genetic studies have demonstrated [17].

In this case, our results support the hypothesis that CYP2R1 is a crucial 25- hydroxylase enzyme. On the other hand, there are discrepancies between the CYP2R1 variants previously associated in different cohorts. In the case of European descent, a number of genome-wide association studies detected more than 25 SNPs in *CYP2R1* linked with vitamin D status [3,4,42,43,44]. The finding that common variants at the *CYP2R1* locus were associated with circulating 25(OH)D represents the strongest evidence to date that CYP2R1 is the enzyme responsible for the critical first step in vitamin D metabolism [3]. In the present study, we found that rs10741657 was associated with serum levels of 25(OH)D in a Spanish population over 65 years old. In accordance, we have found a relationship between haplotype 2 and higher 25(OH)D levels probably due to the combination of rs10741657 allele A in addition to the allele G of rs4588 and allele T of rs6013897 that have been related in previously studies to higher levels of 25(OH)D [19,29]. The prevalence of haplotype 2 was about 19% of our sample, being a considerable percentage.

On the other hand, it is not clear the association between other bone metabolism markers and *CYP2R1* gene. We did not find any correlation with other markers for calcium-phosphate balance (iPTH, albumin, creatinine, total calcium, or phosphorus). Our results are similar to the only previous published study according our knowledge reported by Bjork and cols. explaining that probably total calcium and phosphorus homeostasis parameters are controlled by other different mechanisms [31].

Jiang and cols., in addition to validate *CYP2R1* as risk gene, confirmed the association of a locus containing *CYP24A1* with 25-hydroxyvitamin D concentrations using a large European-ancestry sample size [44]. We have not found any association between variant rs6013897 in *CYP24A1* and total calcium and phosphate homeostasis biomarkers in our population. 

Binding affinities for 25(OH)D vary by DBP isoforms, genetic variants in *GC* explain some of the variability in circulating levels of DBP and 25(OH)D [27]. We found a significant difference in PTH, phosphorus and creatinine concentrations among the *GC* SNPs genotypes. Similar differences were also found among the haplotypes 7 (TGGT) and 4 (TGAA) when four SNPs were combined probably due to the effect of *GC* variants. Among the *GC* SNPs included in haplotypes, the lowest concentrations of PTH, phorsphorus or creatinine were seen in individuals with the allele T of rs4588 and allele G of rs2282679. 

At first, we did not observe any association between 25(OH)D levels and any *GC* analyzed SNP, however, in combination, as we have mentioned before, haplotype 2 (GTAT) was related to higher 25(OH) levels.

We also found an association between haplotype 7 (TGGT) and lower levels of phosphorous. This relationship is probably due to that vitamin D acts stimulating intestinal calcium and phosphorus absorption. There are no previously published studies analyzing phosphorous levels and aforementioned variants.

The strongest association was found between haplotype 4 (TGAA) and lower iPTH levels (*p* < 0.001) and the prevalence was about 2% of the sample.

PTH is a key regulator of calcium balance in the body, and it inversely correlates with 25(OH)D. Increased concentrations of PTH affect bone negatively [45]. PTH has therefore been suggested to be used as a health outcome reference for optimal vitamin D status. However, the threshold values for PTH and 25(OH)D differ considerably among studies hampering this approach.

In accord with our finding, the rs4588 TT genotype had also lower PTH levels and 25(OH)D concentration (no significant) relative to rs4588 GG or rs4588 GT carriers. Our results are similar to the previously published in which rs4588 TT had lower PTH levels [19,29].

Saarnio and cols. hypothesized that free 25(OH)D3 may enter the parathyroid glands and might be transformed to 1,25(OH)2D3 by cytochrome p450 27B1 enzyme (CYP27B1) [29]. The higher amount of 1,25(OH)2D3 could suppress the production of PTH and explains the lower concentration of PTH with the rs4588 TT genotype. On the other hand, other studies reported that total and free 25(OH)D3 were inversely correlated of PTH and the biological effect of vitamin D on PTH level is mainly independent of DBP genotype [32,46]. 

Variability reported values could be explained due to the different fractions analyzed. DBP transports 85–90% of the total circulating 25(OH)D. Although, albumin binds 10–15% and less than 1% of the vitamin D is circulating in its free form, these portions are responsible for its biologic action according the free hormone hypothesis [47,48]. However, the affinity of albumin to 25(OH)D is very weak, so the term bioavailable refers to the circulating 25(OH)D that is not bound to DBP, which is the sum of free and albumin bound fraction [26]. 

Recently, a systematic review of polymorphisms in vitamin D pathway-related genes and vitamin D status showed the highest confirmation rates were found for SNPs in the GC gene rs2282679 (association to vitamin D status confirmed in 77% of the studies); rs4588 (confirmed in 73% of the studies) and in the *CYP2R1* gene rs10741657 (confirmed in 66% of the studies). On the other hand, as further frequently studied SNPs located in the *CYP24A1* gene, rs6013897 SNP was confirmed in 17% of studies [49]. The aforementioned results are in agreement with the differences we have found in our study.

Therefore, a very interesting result is that the genetic variants in *CYP2R1* and *GC* could be predictive of 25(OH)D and iPTH serum levels, respectively, in older Caucasian adults. However, further studies are needed to verify iPTH as a biomarker.

One of the limitations of the study is that we did not analyze free and albumin bounded fraction of 25(OH)D. Variability in serum concentrations of DBP are a major determination of serum 25(OH)D levels, therefore vitamin biological action. However, Peris and cols. established that determination of different forms of 25(OH)D does not offer additional advantages over total 25(OH)D measurement for vitamin D deficiency evaluation [50]. Moreover, we tested vitamin D biological activity by serum PTH in addition to other mineral metabolism biomarkers. Nevertheless, it is also plausible, that in healthy adults, the biological effect of vitamin D on PTH levels seems to be mainly independent of DBP concentrations [28,51,52,53]. On the other hand, we have analyzed a moderate sample size (relative to a study for associations of genetic polymorphisms) and it could be possible that a type II error is being incurred due to the lack of statistical power. Haplotype association analysis found differences in 25(OH)D concentration between women and men in 25(OH)D with just a change in rs6013897, thymine and not adenine. Furthermore, haplotypes 3 and 7 showed differences in phosphorus concentration with the base haplotype in the whole sample and by gender. Those haplotypes differ in rs6013897 for the third haplotype and in rs4588 and rs2282679 for the haplotype 7. Although other haplotypes also shown differences in some studied variables (e.g., haplotype 3 and phosphorus concentration, haplotype 4 and iPTH or haplotype 5 and creatinine), their results could be influenced by sample splitting for this kind of analysis. Since the major frequency of these haplotypes is 9.99% (28 individuals from 284), sample size should be higher to obtain accurate information of that differences and haplotype distribution for future studies.

Finally, our study has several strengths: (1) we focused on an analysis of a healthy population, so biomarkers were not confounded by disease; (2) we evaluated the influence of a complete background of bone mineral serum biomarkers and we also estimated vitamin D biological activity by serum PTH; (3) we measured creatinine, total calcium or phosphorus levels in order to exclude possible pathologies that could have modified vitamin D levels; (4) we selected variants attending previous published GWAS and biological effect; (5) all samples were collected during the same period (from January to May) to homogenize the impact of sun exposure; and (6) the present analyses point to the importance of assessing the joint effects of genes on vitamin D status.

To our knowledge, this is the first study to evaluate the joint association between genetic polymorphism in *CG*, *CYP2R1* or *CYP24A1* and levels of total 25(OH)D and PTH as well as other mineral metabolism biomarkers (albumin, total calcium and phosphorus) in older Spanish population. Moreover, the study follows the STREGA recommendations (STrengthening the REporting of Genetic Association Studies) in order to enhance the transparency of the report, improving the understanding of the role of genetic factors [54].

## 5. Conclusions

Our findings indicate that genetic variants in *CYP2R1* and *GC* are predictive of 25(OH)D and iPTH serum levels, respectively, in healthy older Caucasian adults. The preliminary results suggest the importance of assessing joint effects of genetic variants rather than individual genotypes. Recent reports have suggested the need to analyze the active form of vitamin D in order to better evaluate vitamin D deficiency and also the vitamin D metabolite ratio (VMR) (serum 24,25(OH)2D3/25(OH)D3) has been proposed as a biomarker of vitamin D sufficiency to replace serum 25(OH)D. However, the current study confirmed the role of iPTH as one of the most sensitive biomarkers of vitamin D activity in vivo. Future studies with larger cohorts and more biomarkers are needed to further characterize the joint effects of multiple genes, along with demographic and clinical variables.

## Figures and Tables

**Table 1:** Description of the study cohort.

Characteristics	Overall N = 273	Men *n* = 129	Women *n* = 144	*p*-Value
Age (years)	76.13 (7.09)	76.63 (7.30)	75.65 (6.93)	0.254
BMI (kg/m^2^)	27.61 (3.93)	27.48 (3.68)	27.70 (4.15)	0.639
Current smokers (%)	5.10	5.40	5.30	0.526
Vitamin D supplement (%)	14.80	12.90	16.10	0.276
Sun exposure (%)	64.40	66.90	61.60	0.217
Barthel Index (points)	84.23 (13.68)	84.35 (13.27)	84.01 (14.12)	0.835
MNA (points)	13.89 (1.82)	13.88 (1.80)	13.86 (1.84)	0.930
25(OH)D (ng/mL)	18.40 (8.89)	18.15 (8.31)	18.62 (9.36)	0.665
iPTH (ng/L)	66.84 (31.96)	64.44 (27.38)	67.51 (29.77)	0.385
Total calcium (mg/dL)	9.47 (0.36)	9.40 (0.30)	9.52 (0.37)	**0.005**
Phosphorus (mg/dL)	3.25 (0.50)	3.05 (0.43)	3.42 (0.49)	**<0.001**
Creatinine (mg/dL)	0.93 (0.25)	1.04 (0.23)	0.82 (0.19)	**<0.001**
Albumin (g/dL)	4.42 (0.25)	4.45 (0.25)	4.39 (0.25)	0.05

Note: Values are percentages for categorical data or mean and standard deviation for continuous data. BMI, body mass index; MNA, Mini Nutritional Assessment; 25(OH)D, 25-hydroxivitamin D; iPTH, intact parathyroid hormone.

**Table 2:** Pearson correlation coefficients of analyzed variables.

	Age	BMI	Barthel INDEX	MNA	Creatinine	25(OH)D	Total Calcium	Phosphorus	Albumin	iPTH
age	1 -	−0.085 0.156	−0.263 **<0.001**	−0.057 0.345	0.268 **<0.001**	−0.114 0.062	−0.001 0.989	−0.101 0.099	−0.274 **<0.001**	0.240 **<0.001**
BMI		1 -	0.064 0.288	0.068 0.255	0.043 0.482	0.058 0.341	0.016 0.799	−0.021 0.735	0.086 0.159	−0.010 0.868
Barthel Index			1 -	0.020 0.744	−0.096 0.114	−0.062 0.312	0.096 0.118	−0.085 0.166	0.094 0.196	−0.064 0.298
MNA				1 -	−0.001 0.989	0.054 0.380	−0.081 0.187	−0.126 0.039	0.037 0.548	−0.039 0.531
creatinine					1 -	−0.026 0.667	−0.031 0.616	−0.185 **0.002**	−0.054 0.375	0.222 **<0.001**
25(OH)D						1 -	0.037 0.548	0.133 0.029	0.065 0.298	−0.203 **0.001**
total calcium							1 -	0.052 0.393	0.471 **<0.001**	−0.035 0.575
phosphorus								1 -	−0.007 0.910	−0.090 0.144
albumin									1 -	−0.209 **0.001**
iPTH										1 -

Note: Each cell contains two values: (a) Pearson correlation coefficient; (b) *p* value of testing if the correlation is significant. Statistically significant variables are in bold. Abbreviations: BMI, body mass index; MNA: Mini Nutritional Assessment; 25(OH)D: 25-hydroxivitamin D; iPTH: Intact parathyroid hormone.

**Table 3:** The Hardy–Weinberg equilibrium *p*-values and distribution of genotype frequencies of selected variants.

Gene Variant	Alleles	Major Allele Frequency (%)	*p*, HWE
rs4588	G/T	71.8	0.052
rs2282679	T/G	71.4	0.139
rs10741657	G/A	66.8	0.171
rs6013897	T/A	81.9	0.216

SNP rs4588 was in strong linkage disequilibrium (LD) with rs2282679 (SNAP *R*^2^ = 0.999, D′ = 1).

**Table 4:** Associations between SNPs and bone mineral metabolism biomarkers.

		Crude Model, *p*			Age, *p*-Adjusted			Gender, *p*-Adjusted
Variable	SNP	Codominant	Dominant	Recessive	Codominant	Dominant	Recessive	Codominant
25(OH)D	rs4588	0.115	0.195	0.090	0.206	0.206	0.129	0.357
	rs2282679	0.124	0.198	0.063	0.162	0.210	0.087	0.365
	rs10741657	0.441	0.238	0.374	0.490	0.291	0.366	**0.025**
	rs6013897	0.782	0.636	0.522	0.660	0.526	0.412	0.160
albumin	rs4588	0.156	0.938	0.059	0.359	0.883	0.156	0.285
	rs2282679	0.223	0.789	0.084	0.417	0.733	0.185	0.230
	rs10741657	0.382	0.570	0.329	0.252	0.452	0.268	0.346
	rs6013897	0.341	0.500	0.147	0.550	0.682	0.275	0.803
iPTH	rs4588	0.773	0.719	0.604	0.845	0.603	0.902	**0.026**
	rs2282679	0.781	0.611	0.734	0.780	0.496	0.996	**0.025**
	rs10741657	0.851	0.586	0.727	0.919	0.771	0.706	0.501
	rs6013897	0.902	0.740	0.846	0.754	0.455	0.759	0.823
total calcium	rs4588	0.242	0.586	0.093	0.233	0.528	0.091	0.750
	rs2282679	0.610	0.707	0.324	0.600	0.644	0.323	0.627
	rs10741657	0.704	0.489	0.847	0.753	0.555	0.823	0.488
	rs6013897	0.783	0.936	0.493	0.767	0.878	0.469	0.750
phosphorus	rs4588	0.081	**0.028**	0.927	0.070	**0.027**	0.933	0.626
	rs2282679	0.077	**0.033**	0.832	0.065	**0.032**	0.716	0.197
	rs10741657	0.305	0.131	0.800	0.339	0.147	0.780	0.267
	rs6013897	0.168	0.985	0.074	0.133	0.933	0.053	0.626
creatinine	rs4588	0.070	0.071	0.339	**0.020**	0.054	0.136	**0.009**
	rs2282679	0.069	0.067	0.372	**0.024**	0.052	0.177	**0.009**
	rs10741657	0.104	0.090	0.560	0.139	0.109	0.607	0.587
	rs6013897	0.231	0.184	0.148	0.391	0.269	0.265	0.541

Statistically significant variables are in bold. Abbreviations: 25(OH)D: 25-hydroxivitamin D; iPTH: Intact parathyroid hormone.

**Table 5:** Haplotypes combination.

Haplotype	rs4588	rs2282679	rs10741657	rs6013897	Frequency, %
1	G	T	A	A	4.38
2	G	T	A	T	19.25
3	G	T	G	A	9.99
4	T	G	A	A	2.05
5	T	G	A	T	7.41
6	T	G	G	A	1.77
7	T	G	G	T	16.90
rare	*	*	*	*	0.74
base	G	T	G	T	37.65

* base haplotypes: referent; rare haplotypes: other possible haplotypes with total frequency less than 1%.

**Table 6:** The association of haplotypes with mineral metabolism biomarkers (25(OH)D, iPTH, Phosphorus).

	25(OH)D					iPTH				Phosphorus			
Model *	Haplotype	Coef	SEM	*p* val	AIC	Coef	SEM	*p* val	AIC	Coef	SEM	*p* val	AIC
crude	(Intercept)	19.444	1.370	0.000	1958.4	70.793	4.454	0.000	2629.7	3.411	0.070	0.000	386.94
	1	1.333	2.698	0.622		4.601	7.661	0.549		−0.043	0.124	0.729	
	2	0.238	1.565	0.879		−5.217	4.378	0.235		−0.088	0.073	0.230	
	3	−1.329	1.668	0.426		−4.881	5.290	0.357		−0.176	0.084	**0.036**	
	4	−1.659	4.214	0.694		12.562	1.839	**<0.001**		0.117	0.195	0.549	
	5	−0.561	1.832	0.760		−7.492	6.036	0.216		0.001	0.098	0.992	
	6	−4.705	3.525	0.183		−10.130	11.455	0.377		−0.108	0.206	0.600	
	7	−1.885	1.482	0.204		−0.881	4.848	0.856		−0.269	0.079	**0.001**	
	rare	−1.258	3.716	0.735		−6.199	13.300	0.642		0.137	0.202	0.497	
age	(Intercept)	30.138	6.097	0.000	1950.8	−10.539	2.369	0.000	2602.8	3.884	0.337	0.000	386.94
	1	0.492	3.573	0.890		8.836	8.060	0.274		−0.067	0.127	0.598	
	2	0.443	1.851	0.811		−5.123	4.210	0.225		−0.081	0.073	0.269	
	3	−1.282	1.870	0.493		−3.378	4.979	0.498		−0.177	0.084	**0.035**	
	4	−0.841	4.941	0.865		8.526	14.595	0.560		0.137	0.192	0.475	
	5	−0.725	1.906	0.704		−6.881	6.028	0.255		−0.004	0.099	0.971	
	6	−5.013	3.504	0.154		−7.392	11.458	0.519		−0.117	0.206	0.571	
	7	−1.651	1.529	0.281		−1.818	4.688	0.699		−0.263	0.080	**0.001**	
	rare	−1.824	3.726	0.625		−1.328	12.664	0.917		0.113	0.203	0.579	
gender	(Intercept)	20.802	1.889	0.000	1957.000	66.061	3.844	0.000	2638.2	3.237	0.102	0.000	346.16
	1	0.203	4.344	0.963		5.089	3.191	0.112		−0.183	0.254	0.471	
	2	6.464	2.438	**0.009**		7.490	6.365	0.240		0.069	0.133	0.602	
	3	4.649	3.005	0.123		3.050	7.398	0.680		0.068	0.159	0.670	
	4	12.269	6.176	0.048		−33.469	7.713	**<0.001**		−0.238	0.319	0.457	
	5	−1.534	3.536	0.665		−8.434	10.721	0.432		0.565	0.181	**0.002**	
	6	0.820	6.807	0.904		−16.115	5.500	0.004		0.475	0.418	0.257	
	7	0.826	2.730	0.762		−11.411	6.850	0.097		−0.028	0.147	0.850	
	rare	0.359	9.825	0.971		−30.353	6.777	**<0.001**		−0.053	0.497	0.915	

*: crude model, adjusted by age, adjusted by gender. Statistically significant variables are in bold. Abbreviations: 25(OH)D: 25-hydroxivitamin D; iPTH: Intact parathyroid hormone. SEM: standard error of the mean.

**Table 7:** The association of haplotypes with mineral metabolism biomarkers (Albumin, total Calcium and Creatinine).

	Albumin					Total Calcium				Creatinine			
Model *	Haplotype	Coef	SEM	*p* val	AIC	Coef	SEM	*p* val	AIC	Coef	SEM	*p* val	AIC
crude	(Intercept)	4.401	0.037	0.000	40.69	9.472	0.054	0.000	229.86	0.956	0.036	0.000	25.11
	1	0.074	0.064	0.249		0.024	0.090	0.790		−0.024	0.061	0.693	
	2	0.019	0.037	0.619		0.005	0.055	0.925		−0.052	0.035	0.141	
	3	0.043	0.044	0.331		0.039	0.062	0.529		−0.047	0.040	0.243	
	4	−0.095	0.096	0.320		−0.135	0.140	0.335		−0.102	0.096	0.287	
	5	−0.012	0.052	0.811		−0.029	0.076	0.707		0.051	0.054	0.344	
	6	0.047	0.117	0.688		−0.028	0.164	0.864		−0.075	0.110	0.495	
	7	0.003	0.042	0.947		−0.013	0.062	0.839		0.006	0.040	0.873	
	rare	0.049	0.106	0.648		0.171	0.152	0.260		−0.036	0.103	0.724	
age	(Intercept)	5.127	0.170	0.000	24.59	9.374	0.249	0.000	229.54	0.009	0.002	0.000	9.54
	1	0.045	0.063	0.480		0.027	0.091	0.762		0.007	0.060	0.906	
	2	0.024	0.038	0.524		0.006	0.055	0.906		−0.052	0.034	0.122	
	3	0.041	0.044	0.356		0.042	0.062	0.500		−0.039	0.040	0.325	
	4	−0.071	0.097	0.463		−0.139	0.137	0.314		−0.131	0.093	0.163	
	5	−0.016	0,052	0.756		−0.023	0.075	0.760		0.051	0.051	0.321	
	6	0.012	0.127	0.925		−0.015	0.162	0.924		−0.061	0.109	0.576	
	7	0.012	0.045	0.782		−0.020	0.062	0.747		0.007	0.039	0.863	
	rare	0.010	0.103	0.921		0.179	0.152	0.240		0.001	0.101	0.992	
gender	(Intercept)	4.430	0.057	0.000	40.66	9.381	0.078	0.000	232.83	1.079	0.047	0.000	−37.29
	1	−0.237	0.121	0.052		−0.281	0.170	0.100		−0.012	0.103	0.909	
	2	−0.003	0.071	0.969		−0.031	0.106	0.768		0.061	0.059	0.299	
	3	0.023	0.087	0.788		0.007	0.120	0.954		0.059	0.071	0.409	
	4	0.420	0.250	0.093		0.154	0.346	0.657		−0.019	0.163	0.907	
	5	−0.045	0.099	0.652		0.030	0.147	0.837		−0.214	0.086	**0.014**	
	6	0.212	0.224	0.346		0.112	0.312	0.720		−0.055	0.195	0.777	
	7	0.031	0.086	0.719		−0.054	0.120	0.650		−0.049	0.067	0.458	
	rare	−0.253	0.283	0.371		0.282	0.402	0.484		0.266	0.243	0.274	

*: crude model, adjusted by age, adjusted by gender. Statistically significant variables are in bold.

## Abbreviations

ADL	Activities of daily life
AIC	Akaike Information Criterion
BI	Barthel index
BMI	Body mass index
CGR	Clinical Group Risk
CYP24A1	Cytochrome P450 family 24 subfamily A member 1
CYP2R1	Cytochrome P450 Family 2 Subfamily R Member 1
DBP	Vitamin D–binding protein
GC	Vitamin D–binding protein gene
1,25(OH)2D	1,25-Dihydroxyvitamin D
25 (OH)D	25-Hydroxyvitamin D
IOM	Institute of Medicine
iPTH	Intact parathyroid hormone
MMSE	Mini Mental State Examination
MNA	Mini Nutritional Assessment
SD	Standard deviation
SEM	Standard error of the mean
